# Circulating Unmetabolized Folic Acid: Relationship to Folate Status and Effect of Supplementation

**DOI:** 10.1155/2012/485179

**Published:** 2012-02-19

**Authors:** Carolyn Tam, Deborah O'Connor, Gideon Koren

**Affiliations:** The Motherisk Program, Division of Clinical Pharmacology/Toxicology and Nutrition Sciences, The Hospital for Sick Children, Toronto, ON, Canada M5G 1X8

## Abstract

There are increasing concerns that exposure to unmetabolized folic acid, which results from folic acid intakes that overwhelm the liver's metabolic capacity, may be associated with adverse effects. In this paper, we examined the folic acid status of women of reproductive age in relation to dietary intake and the effect of folic acid supplementation (1.1 mg or 5 mg). Plasma unmetabolized folic acid was not significantly correlated with folate intake estimated by food frequency questionnaire or biomarkers. The proportion of women with detectable levels of unmetabolized folic acid increased from 65% to 100% after twelve weeks of supplementation (*P* < 0.05); however, the increase in concentrations did not reach statistical significance and the effect was not sustained. Moreover, there were no significant differences between the two doses. This suggests that there are mechanisms by which the body adapts to high folic acid intakes to limit exposure to unmetabolized folic acid.

## 1. Introduction

**Figure 1:**
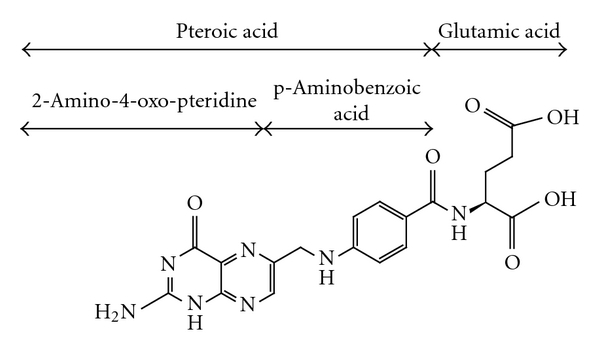
Structure of folic acid [1]. The three parts of the parent structure include 2-amino-4-oxo-pteridine, p-aminobenzoic acid, and glutamic acid. Substitutions (one-carbon groups) occur at the N5 and/or N10 positions.

The term “folate” describes the group of B vitamers that share the same vitamin activity based on the parent structure of folic acid. The parent structure consists of an aromatic pteridine ring joined by a methylene bridge to para-aminobenzoic acid (PABA), which in turn is attached to glutamic acid by a peptide bond (Figure 1) [1]. Folate vitamers differ in the oxidation state of the pteridine ring and substitution on the N5 and/or N10 nitrogen atoms. In addition, a polyglutamate tail consisting of up to nine glutamate residues, each one joined via amide linkage to the *γ*-carboxyl group of the preceding residue, may be added [1, 2]. 

By convention, the term “folic acid” refers specifically to the fully oxidized and most stable form of the vitamin that is used in supplements and fortified foods.

Biologically, these groups of vitamins are critical in DNA synthesis and repair, and as cofactors in biological reactions involving folate sources. Folate exists naturally in foods as reduced folate polyglutamate conjugates. In addition, folic acid is added as a fortificant to certain foods. As of 2007, fifty-two countries worldwide had national regulations mandating folic acid fortification of wheat flour [3]. Folic acid is also found in supplements and multivitamins.

In general, the bioavailability of folic acid is higher than that of the naturally occurring food folates. Under fasting conditions, the bioavailability of folic acid approaches 100% [4]. When consumed with food, folic acid (either supplemental or as a fortificant) is about 85% bioavailable [4]. The bioavailability of naturally occurring food folates depends on whether folate is present primarily as a monoglutamate or polyglutamate (with the former being more bioavailable) and the presence or absence of dietary and nondietary factors that can facilitate or inhibit folate absorption; on average, it is estimated to be 50%, although it can be as high as 60 to 90% from some fruits and vegetables [5].

Neural tube defects (NTDs) are congenital malformations produced by failure of the neural tube to form and close properly during embryonic development. The most common types of NTDs include spina bifida and anencephaly [6]. 

A relationship between folate deficiency and malformations of the central nervous system was first suggested in the 1960s by Smithells et al. [7]. 

To clarify the issue, the Medical Research Council of the United Kingdom initiated a multicentre, double-blind, randomized controlled trial (the “MRC Vitamin Study”) to evaluate the effect of folic acid with or without other vitamins on the rate of NTD recurrence among women who had a previous pregnancy complicated by an NTD [8]. Among 1195 informative pregnancies, the prevalence of NTDs among women allocated to receive folic acid was 1.0%, compared to 3.5% among women in the other groups. Thus folic acid reduced the risk of NTD recurrence by 72% (relative risk, RR, 0.28; 95% confidence interval (CI), 0.12–0.71). The effect of the “other vitamins” on the risk of NTD recurrence was nonsignificant (RR 0.80; 95% CI, 0.32–1.72).

In parallel to the MRC Vitamin Study, a single-centre, double-blind, randomized controlled trial (the “Hungarian randomized controlled trial”) was conducted to evaluate the effect of multivitamin supplementation on first occurring NTDs. There were no NTDs among 2104 pregnancies in the multivitamin/mineral group (*P* = 0.029). The protective effect of the multivitamin (containing 0.8 mg of folic acid) on first occurrent NTDs was estimated to be 90% [9].

In contrast to natural food folates, folic acid is a non-coenzymatic form of folate. Folic acid metabolism involves conversion of folic acid to coenzymatic tetrahydrofolate derivatives, primarily 5-CH_3_-H_4_PteGlu. The first and rate-limiting step is catalyzed by DHFR, which reduces folic acid to H_2_PteGlu (and subsequently to H_4_PteGlu) [10]. The expression and activity of DHFR in human liver is relatively low, such that oral doses greater than 260 to 280 *μ*g can saturate the hepatic metabolic capacity, resulting in the appearance of “unmetabolized” folic acid in plasma [11–13]. With single doses, however, folic acid is rapidly cleared from plasma through a combination of uptake into peripheral tissues and renal excretion [14]. On the other hand, two studies have demonstrated the presence of detectable levels of unmetabolized folic acid in fasting plasma samples after eight to 14 weeks of supplementation with 400 *μ*g/day of folic acid [15, 16]. This suggests that daily ingestion of more than 400 *μ*g of folic acid saturates not only hepatic DHFR activity, but also cellular uptake and renal clearance mechanisms.

There is much debate as to whether exposure to unmetabolized folic acid poses a health risk [17]. Theoretically, folic acid could interfere with normal folate metabolism through competition with reduced, coenzymatic folates for transporters, binding proteins, and folate-dependent enzymes [18–20]. For instance, both folic acid and H_2_PteGlu are substrates for DHFR. Although the affinity of DHFR for H_2_PteGlu is higher than its affinity for folic acid, in the presence of high concentrations of folic acid, folic acid could competitively inhibit the conversion of H_2_PteGlu to H_4_PteGlu [19]. As neither folic acid nor H_2_PteGlu is metabolically active, this could theoretically create an intracellular folate deficiency [19]. Another study observed a downregulation of folate transporters in intestinal and renal epithelial cells cultured in growth media that were oversupplemented with folic acid [20]. Although it remains to be seen whether these *in vitro* effects also occur *in vivo*, the potential implications of disturbed folate metabolism are wide-ranging; it is, therefore, critical that we gain a better understanding of the pharmacokinetics and pharmacodynamics of unmetabolized folic acid.

At present, there is no conclusive evidence that exposure to unmetabolized folic acid causes adverse health effects. However, potential concerns (i.e., those that appear to be uniquely associated with high folic acid intakes) include negative effects on vitamin B12 deficiency, cancer development, immune function, and epigenetic regulation [21].

The objectives of the present study were twofold.

1. To examine the relationship between plasma concentration of unmetabolized folic acid and (a) dietary folic acid and total folate intake and (b) plasma and red blood cell total folate concentration.
2. To examine the effect of folic acid supplementation on fasting plasma concentrations of unmetabolized folic acid.

## 2. Materials and Methods

### 2.1. Study Population

The blood samples and dietary data presented herein were collected as part of a prospective, randomized, open-label trial of 30 weeks of daily supplementation with 1.1 mg or 5 mg of folic acid as part of a prenatal/postpartum vitamin-mineral supplement [22]. Participants were recruited through posters displayed at the Hospital for Sick Children (Toronto, ON) and at designated locations on the St. George campus of the University of Toronto, advertisement in the Hospital for Sick Children newsletter, online advertisements published on the Hospital for Sick Children and Motherisk websites, and word-of-mouth. Motherisk is a clinical, research, and teaching program affiliated with the University of Toronto that provides evidence-based information on the safety of medications, infections, and other exposures during pregnancy and lactation.

All participants were healthy women between the ages of 18 and 45 at the time of study enrollment. Eligibility was assessed during an initial telephone or in-person interview. Potential participants were screened for the following conditions and excluded from participation, as appropriate.

- Use of folic acid supplements or multivitamin supplements containing folic acid in the six months preceding study enrollment.
- Previous pregnancy in which an NTD was detected
- Family history of NTDs.
- Concurrent use of medications known to affect folate status (e.g., antiseizure medications, folate antagonists, and oral contraceptives).
- Allergy or hypersensitivity to any of the ingredients in PregVit or PregVit-Folic5.

The protocol for the trial was approved by the Research Ethics Board at The Hospital for Sick Children. Eligible participants provided *verbal* informed consent to proceed with study enrollment; enrolled participants provided *written* informed consent at the first clinic appointment. Information collected during the enrollment process included:

- contact information,
- demographic information (e.g., ethnicity, marital status, education, and employment status),
- history of medication and substance use,
- medical and obstetrical histories.

Randomization was performed by the Hospital for Sick Children research support pharmacists. Participants were randomized to receive either PregVit (containing 1.1 mg of folic acid) or PregVit-Folic5 (containing 5 mg of folic acid). Neither the participants nor the study coordinator were blinded, as it was not feasible to modify the product appearance or packaging.

### 2.2. Study Drugs

PregVit and PregVit-Folic5 are vitamin-mineral supplements designed for use by planning and pregnant women. Both PregVit and PregVit-Folic5 are formulated as two tablets that are to be taken daily: the pink (am) tablet is taken in the morning and the blue (pm) tablet in the evening. The pink tablet and blue tablet contain different vitamins and minerals (Table 1); specifically, iron is supplied in the pink tablet and calcium is supplied in the blue tablet to facilitate iron absorption and reduce adverse events related to iron supplementation.

### 2.3. Study Procedures

Study visits were conducted in the Clinical Investigations Unit at the Hospital for Sick Children. All participants provided witnessed, written informed consent at the beginning of the first study visit.

At the first study visit, participants provided a fasting blood sample (5 mL) to measure baseline plasma and RBC folate and plasma vitamin B12. Participants were given an eight-week supply of their assigned multivitamins, which was renewed at subsequent study visits, and instructed to take the multivitamins as per the product monograph (i.e., pink tablet in the morning, blue tablet in the evening). Further instruction was given to leave missed or skipped doses in the blister packaging and to return all packaging, including unused tablets, to the study coordinator. Rates of adherence were determined based on the number of pills returned. Participants returned to the hospital at weeks 2, 4, 6, 12, and 30 (±3 days) to provide fasting blood samples (5 mL) for plasma and RBC folate measurements. Plasma vitamin B12 was measured again at week 30.

A validated FFQ [23] was administered during the first and final study visits to assess usual dietary folate intake during the six months prior to study participation and during the 30 weeks (approximately seven months) of study participation, respectively.

### 2.4. Blood Folate Analyses

#### 2.4.1. Blood Sample Preparation

Venous blood samples were collected in ethylenediaminetetraacetic acid- (EDTA-) treated blood collection tubes (BD Vacutainer K2 EDTA; BD Biosciences, Franklin Lakes NJ) after a minimum six-hour fast. Samples were shielded from light, placed near ice, and processed within two hours of collection.

To determine the hematocrit (Hct), whole blood was drawn into 75 mm heparinized capillary tubes (Allied Corp., Fisher Scientific; Pittsburgh PA) and centrifuged for 3 minutes (Hettich Haematokrit; Tuttlingen, Germany). Hct was reported as the mean of at least two determinations.

Whole blood samples were prepared in triplicate in 2 mL polypropylene microtubes (Sarstedt, Inc., Montréal, QC). Aliquots (100 *μ*L) of EDTA-anticoagulated whole blood were diluted 10-fold in 1% (wt : vol) ascorbic acid (A7631; Sigma-Aldrich Canada Ltd.; Oakville, ON) in deionized water. Samples were vortexed and incubated at 37°C for 30 minutes to allow for lysis of red blood cells and deconjugation of polyglutamylated folates by plasma GGH.

The remaining whole blood was centrifuged at 1500 g for 20 minutes at 4°C (Allegra 21R Centrifuge; Beckman Coulter, Inc., Fullerton, CA) to separate plasma from RBCs. The plasma layer was removed to a 14 mL polypropylene tube (Falcon; BD Biosciences, Franklin Lakes NJ). Aliquots (500 *μ*L) of plasma were transferred to two 2 mL polypropylene microtubes for vitamin B12 analysis. Sodium ascorbate (134032; Sigma-Aldrich Canada Ltd.; Oakville, ON) was added to the remaining plasma to a final concentration of 1% (wt : vol) to prevent oxidative degradation of folate.

All samples were frozen immediately after processing and stored at −80°C.

#### 2.4.2. Affinity-HPLC Assay for Oxidized Folic Acid

Plasma concentrations of oxidized folic acid were measured by the affinity-HPLC method with electrochemical detection described by Bagley and Selhub [24] and Belz and Nau [25].

The affinity column consisted of immobilized FBP that was isolated from dried whey powder. To isolate FBP, 50 g of dried whey powder (ADM Nutraceuticals; Decatur, IL) was suspended in 500 mL of water and the pH was adjusted to pH 9 with 5 mol/L sodium hydroxide. The suspension was refrigerated overnight and then centrifuged at 10 000 g for 30 minutes at 4°C. To prepare the column matrix, the supernatant fraction was allowed to react with Affi-Prep 10 affinity chromatography support (Bio-Rad Laboratories; Mississauga, ON) overnight at 4°C. The FBP-Affi-Prep 10 slurry was washed sequentially with 20 mmol/L trifluoroacetic acid, 1 mol/L potassium phosphate, and deionized water. To prepare the column, 1 mL of the slurry was transferred to a glass Pasteur pipette packed with glass wool. Folate recovery from the prepared column, determined using [^3^H]-folic acid (Amersham Biosciences; GE Healthcare; Piscataway, NJ), was 95 ± 2% (*n* = 10). 

To prepare the samples for HPLC analysis, frozen plasma samples were placed in a water bath set at 100°C for 10 minutes to denature plasma proteins. Denatured samples were loaded on the prepared FBP-Affi-Prep 10 column, which was washed sequentially with deionized water to remove nonfolate compounds and then mobile phase (equal proportions of A, B, and C; described in further detail below) to elute the purified folates.

Folic acid was quantified in affinity-purified samples by reversed-phase HPLC with electrochemical detection. The HPLC system consisted of a low-pressure gradient pump (P580A LPG) fitted with an automated sample injector (ASI-100 Autosampler) set at 4°C to minimize sample degradation, phenyl analytical column (250 mm × 4.6 mm internal diameter, 5 *μ*m particle size; BetaSil* Phenyl HPLC Column) installed in an oven set at 30°C (STH 585), and ED50 electrochemical detector with Ag/AgCl reference electrode managed by a computer running Chromeleon software (Version 6.2). The analytical column was purchased from Keystone Scientific (Thermo Fisher Scientific, Inc., Waltham, MA). All other parts and software were purchased from Dionex Corp. (Oakville, CA).

The mobile phase was delivered at a flow rate of 0.75 mL/min and maintained at 25% A (112 mmol/L potassium phosphate, 240 mmol/L phosphoric acid), 7% B (80% (vol : vol) acetonitrile in HPLC-grade water), and 68% C (HPLC-grade water) for the first 10 minutes. Between 10 and 40 minutes, the concentration of B was raised linearly to 20%, providing the gradient. The folic acid derivative was identified on the basis of retention time and comparison to the electrochemical response of the peak of the folic acid standard (F8798; Sigma-Aldrich Canada Ltd.; Oakville, ON).

The limit of detection (signal-to-noise ratio = 3) of our HPLC setup was 100 pg of folic acid. Samples for which measured values were below the limit of quantification were spiked with a known quantity of folic acid standard and reassayed.

Assay performance was evaluated using the standard curve that was generated at the beginning of each assay by injecting the folic acid standard in increasing volumes within the linear range of the assay. Injector precision and retention time reproducibility were within the specified limits (relative standard deviation ≤ 1%).

#### 2.4.3. Microbiological Assay for Total Folate

Plasma and whole blood total folate concentrations were measured using the microtitre plate method described by Molloy and Scott [26], with modification, using the test organism *Lactobacillus rhamnosus* (ATCC 7469; American Type Culture Collection, Manassas, VA), which was reconstituted (thawed) daily from a cryopreserved stock.

To prepare the assay medium, 5.7 g of dehydrated assay medium (Difco Folic Acid Casei Medium; BD Biosciences, Franklin Lakes, NJ) was reconstituted in 100 mL of deionized water. Ascorbic acid was added to a final concentration of 0.05% (wt : vol) and the mixture was heated. When the mixture was hot, but not boiling, 30 *μ*L of Tween 80 (P8074; Sigma-Aldrich Canada Ltd.; Oakville, ON) was added and the mixture was brought to the boil for 2 to 3 minutes. After cooling slightly, 0.075 mg of ascorbic acid was added. The medium was shielded from light and held in an incubator at 37°C while the standards and test samples were prepared and deposited. When ready, thawed *L. rhamnosus* suspension (20 *μ*L) was added to 50 mL of assay medium.

Frozen plasma and whole blood samples were thawed at room temperature, shielded from light. Thawed samples were diluted 80-fold in 1% (wt : vol) sodium ascorbate in deionized water. Aliquots of 20, 40, and 60 *μ*L were deposited in triplicate. 1% sodium ascorbate was added to a volume of 100 *μ*L followed by 200 *μ*L of prepared assay medium.

Microtitre plates were covered with aluminum sealing tape (Corning; Sigma-Aldrich Canada Ltd.; Oakville, ON) and incubated at 37°C for 42 hours (Revco Ultima; Thermo Fisher Scientific, Inc., Waltham, MA). After 42 hours, the plates were inverted and agitated to resuspend the cells, and the sealing tape was removed. The optical density at 590 nm was determined using a 96-well microtiter plate reader (Opsys MR; DYNEX Technologies; Chantilly, VA) linked to a computer running the supplied software for data collection and analysis (Revelation Quicklink; DYNEX Technologies; Chantilly, VA). Folate concentrations were determined based on the standard curve that was generated for each plate. The standard curve was based on a folic acid standard (0.5 ng/mL) that was prepared daily from a frozen stock and deposited in triplicate in aliquots of 0 to 100 *μ*L (0 to 50 pg of folic acid).

All samples for a given subject were analyzed as a set to reduce intraperson variability. Measurements were discarded if the coefficient of variation for the triplicate exceeded 5% or if the measurements did not fall in linear range of the standard curve (7–21 pg); if no usable values remained for one or more weeks from a given subject, all samples from that subject were reassayed, adjusting the dilutions as needed. Otherwise, reported values (in picograms) were plotted against the volume of the initial aliquot (i.e., 20, 40, and 60 *μ*L); the slope of the line of best fit gave the folate concentration in the reaction well, which was multiplied by the dilution factor to determine the folate concentration in the plasma or whole blood sample. RBC folate was calculated according to the following equation:

(1) $$\begin{array}{cc} & \text{RBC}\;\text{folate}\\ & \;\;=\;\frac{\text{whole}\;\text{blood}\;\text{folate}-\left[\left(1-\text{Hct}\right)\times \text{plasma}\;\text{folate}\right]}{\text{Hct}}. \end{array}$$

Assay performance (accuracy and interassay variability) was assessed using a certified whole blood folate standard (95/528; National Institute for Biological Standards and Control, Hertfordshire, UK) that was analyzed in triplicate at two different dilutions on each plate. Reported values were checked against a quality control chart prepared in advance from twenty consecutive assays in which the standard was similarly analyzed. The acceptable limits were defined as ±1 standard deviation from the mean of these twenty determinations. Our analyses yielded an overall interassay coefficient of variation of 3.4% and a measured concentration of 30.6 ± 1.0 nmol/L (stated value: 29.5 nmol/L; [27]).

### 2.5. Dietary Folate Analyses

The Block Folic Acid/Dietary Folate Equivalents (DFE) Screener (NutritionQuest; Berkeley CA) was administered to assess dietary folate intake. The DFE Screener is an abbreviated folate-targeted food and supplement screening tool that was developed based on dietary data from NHANES 1999-2000 and designed to assess usual and customary folate intake in women [27–29]. It includes 19 food groups and two supplement questions.

Questionnaires were processed and analyzed by NutritionQuest (Berkeley, CA). The results were reported as:

- naturally occurring food folates (*μ*g),
- folic acid from folic acid-fortified foods (*μ*g),
- total food folate, *μ*g (sum of (a) and (b)),
- total food folate, *μ*g DFE (*μ*g DFE = (a) + (b) × 1.7).

### 2.6. Statistical Analyses

Data were tested for normality using the Shapiro-Wilk test, and parametric or nonparametric tests were performed as appropriate. All statistical analyses were performed using SAS for Windows (Version 9.1; SAS Institute, Inc.; Cary NC), except for the Friedman test due to the lack of posthoc analysis options in SAS. Results were considered statistically significant at a *P* value of ≤0.05.

Subject characteristics are presented as mean ± standard deviation or median (range). Between-group comparisons were performed using Student's *t*-test, Wilcoxon-Mann-Whitney test, or Fisher's exact test. Dietary folic acid intake and dietary total folate intake were compared between groups and over time by using the MIXED procedure (PROC MIXED) in SAS for Windows. The MIXED procedure fits mixed linear models to data and estimates and tests the significance of between- and within-subject effects.

The relationships between plasma unmetabolized folic acid and (i) dietary folic acid intake, (ii) dietary total folate intake, (iii) total plasma folate concentration, and (iv) total RBC folate concentration were evaluated by calculating Kendall's tau-b rank correlation coefficient, which measures association based on concordance and discordance between paired observations. Kendall's tau-b was seen as preferable to Spearman's rho due to the number of tied ranks (i.e., samples that were below the LOD).

Frequency data, including the proportion of women with detectable concentrations of unmetabolized folic acid, were analyzed by Fisher's exact test for between-group comparisons or Cochran's *Q* test for within-group comparisons (i.e., change over time). A significant *Q* statistic was investigated further by planned posthoc pair-wise comparisons using McNemar's test with Bonferroni correction for multiple testing to maintain a procedure-wise type I error rate of 0.05.

As unmetabolized folic acid concentrations were not normally distributed and it was not feasible to transform the data to fit a normal distribution, the effect of folic acid supplementation on plasma folic acid was analyzed using the Friedman test in WINKS SDA 6.0 (TexaSoft; Cedar Hill TX). A significant *χ* ^2^ statistic was investigated further by nonparametric posthoc pair-wise comparisons with Tukey adjustment for multiple testing.

## 3. Results

### 3.1. Study Population

**Figure 2:**
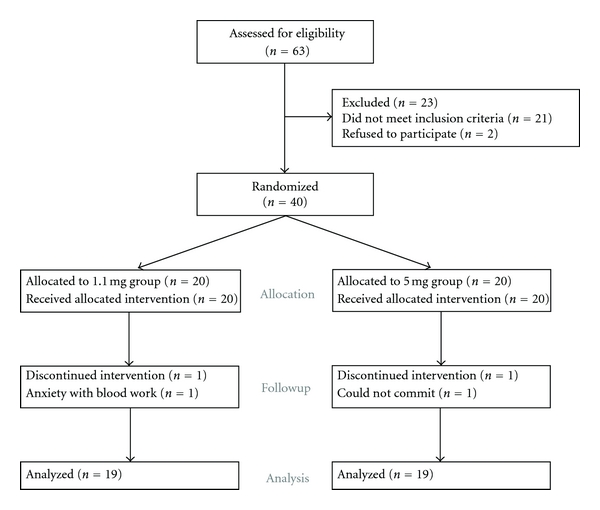
Consolidated Standards of Reporting Trials (CONSORT) patient flow diagram.

Between March 2007 and February 2008, sixty-three healthy, nonpregnant women of reproductive age were approached for participation in this study (Figure 2). Twenty-three women were excluded, either because they did not meet the inclusion criteria (*n* = 21) or because they did not wish to participate (*n* = 2); thus 40 women were enrolled. Twenty women were randomized to take the multivitamin containing 1.1 mg of folic acid; twenty women were randomized to take the multivitamin containing 5 mg of folic acid. One woman from each group withdrew from the study after the baseline measurement due to anxiety with the blood work (*n* = 1) or inability to commit to the study timeline (*n* = 1). Nineteen women in each group completed the study protocol and were included in the analyses.

There were no significant differences between the two groups of women in the collected patient characteristics (Table 2). Except for one woman who was a student pursuing postsecondary education, all of the women had earned a postsecondary degree (either college or university). The majority of the women were employed—either part time or full time. As per the inclusion criteria, all participants were healthy and were not taking any medications on a chronic basis. In the 1.1 mg folic acid group, one woman used acetaminophen or ibuprofen for infrequent migraine headaches and one used minocycline for acne on an “as needed” basis. In the 5 mg folic acid group, two women reported occasional use of salbutamol for asthma and one received desensitization shots for seasonal allergies. Occasional (social) alcohol consumption was reported by the majority of the women and did not differ significantly between the two groups. One woman in the 5 mg group reported light cigarette smoking.

### 3.2. Relationship between Plasma Folic Acid and Other Indicators of Folate Status

The relationships between plasma concentration of unmetabolized folic acid and dietary folate intakes and between plasma folic acid and total blood folate concentrations were evaluated by calculating Kendall's tau-b rank correlation coefficient. Correlation coefficients were calculated within each group and for pooled data from both groups, as these analyses were performed on baseline data (i.e., samples collected before supplementation was started).

**Figure 3:**
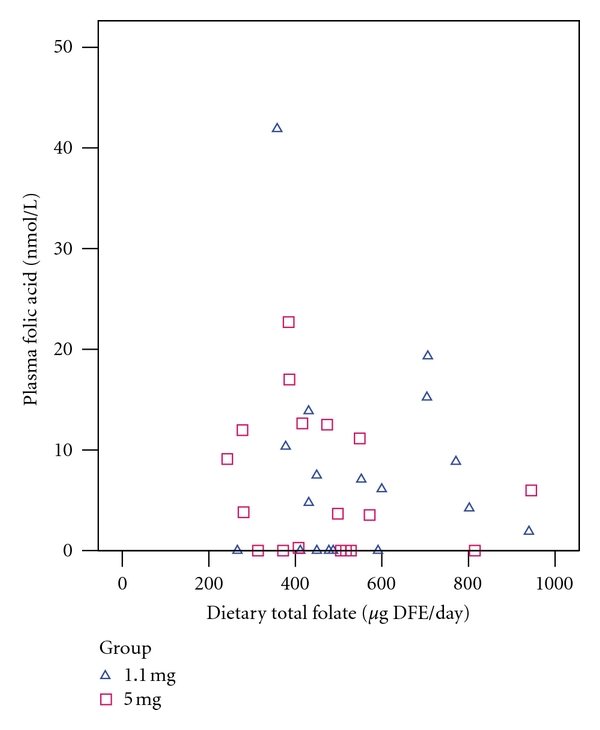
Relationship between plasma folic acid and dietary folic acid intake. 1.1 mg group (triangles): Kendall's *τ*_b_ = 0.12, *P* = 0.47. 5 mg group (squares): Kendall's *τ*_*b*_ = −0.18, *P* = 0.29. Pooled: Kendall's *τ*_*b*_ = −0.026, *P* = 0.83.

**Figure 4:**
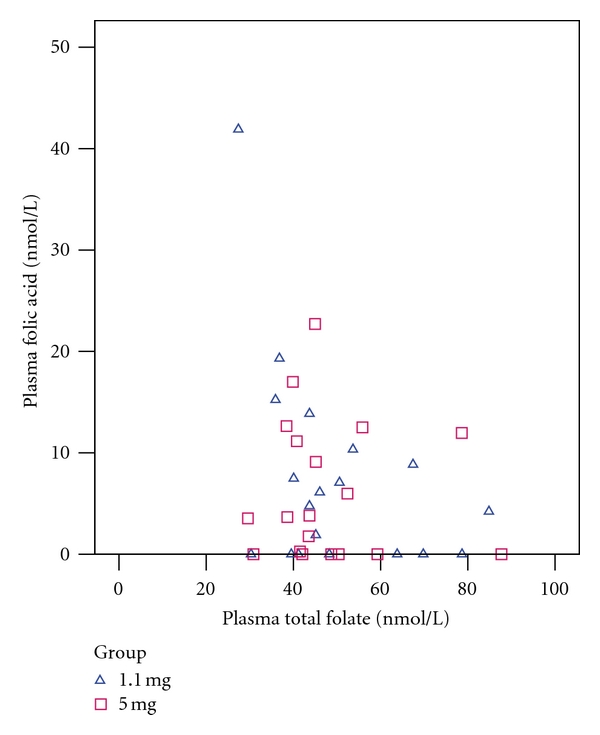
Relationship between plasma folic acid and plasma total folate. 1.1 mg group (triangles): Kendall's *τ*_*b*_ = −0.26, *P* = 0.14. 5 mg group (squares): Kendall's *τ*_*b*_ = −0.073, *P* = 0.67. Pooled data: Kendall's *τ*_*b*_ = −0.16, *P* = 0.18.

**Figure 5:**
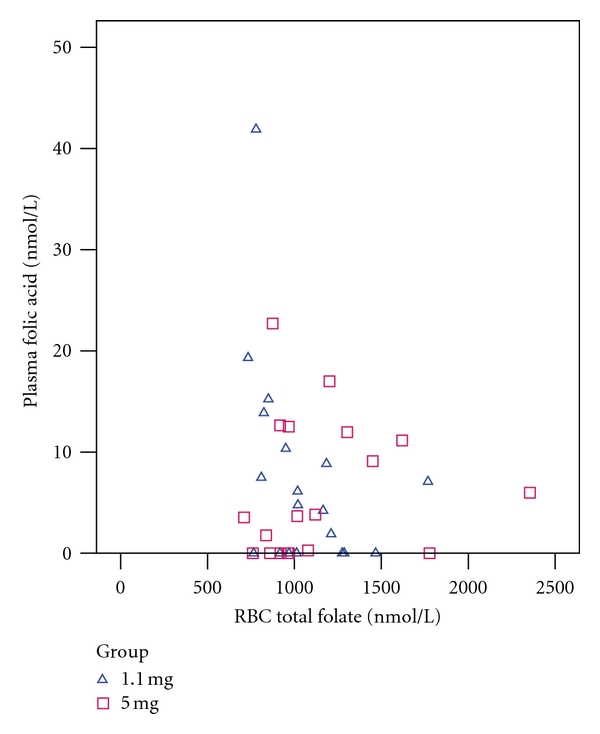
Relationship between plasma folic acid and RBC total folate. 1.1 mg group (triangles): Kendall's *τ*_*b*_ = −0.36, *P* = 0.04. 5 mg group (squares): Kendall's *τ*_*b*_ = 0.15, *P* = 0.39. Pooled data: Kendall's *τ*_*b*_ = −0.068, *P* = 0.56.

Plasma folic acid was not found to be significantly correlated with dietary folic acid or dietary total folate (Figure 3) when analyzed by group or in pooled data. A significant negative correlation was observed between plasma folic acid and RBC total folate in the 1.1 mg group (Kendall's *τ*_*b*_ = −0.36, *P* = 0.04), but not in the 5 mg group or in pooled data. After Bonferroni correction for multiple testing, however, the correlation did not retain statistical significance (critical *P* value = 0.05 ÷ 12 = 0.004). No other significant correlations were observed between plasma folic acid and plasma total folate (Figure 4) or RBC total folate (Figure 5).

Subjects were then grouped according to whether or not unmetabolized folic acid was detectable at baseline and compared on the same dietary and biochemical variables (Table 3). Neither dietary folic intake nor dietary total folate intake was significantly higher among individuals with detectable folic acid compared to those with undetectable levels; similarly, neither plasma nor RBC total folate concentrations were significantly different between the two groups.

### 3.3. Effect of Folic Acid Supplementation on Plasma Concentrations of Unmetabolized Folic Acid

#### 3.3.1. Adherence to Multivitamin Supplementation

Adverse events were reported by fourteen women (37%) over the course of 30 weeks of multivitamin supplementation, including nausea (*n* = 5), constipation (*n* = 3), abdominal discomfort (*n* = 3), diarrhea (*n* = 1), difficulty swallowing (*n* = 1), and heartburn (*n* = 1). All adverse events were mild in nature, however, and none of the women discontinued supplementation or withdrew from the study as a result of the event. Moreover, there was no significant difference in adverse events between the two groups.

**Figure 6:**
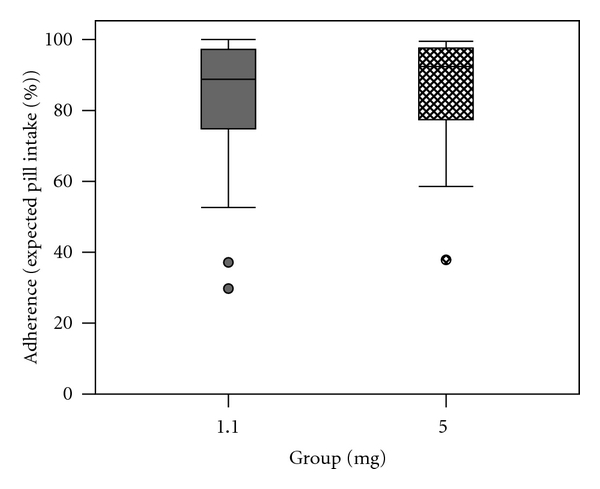
Rates of adherence to multivitamin supplementation.

The median rate of adherence was 88.8% (29.8–100%) in the 1.1 mg group and 89.8% (range 37.9–99.5%) in the 5 mg group (Figure 6). The difference was not significant (*z* = −0.37; *P* = 0.71).

#### 3.3.2. Proportion of Plasma Samples with Detectable Folic Acid

**Figure 7:**
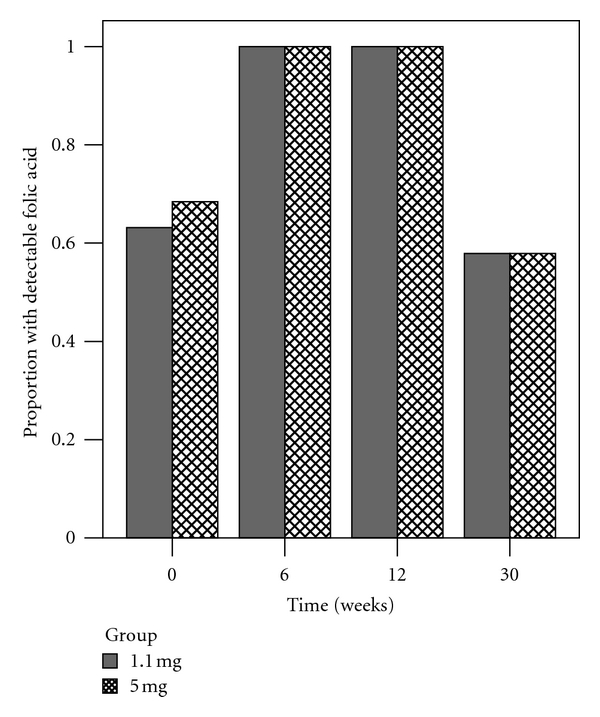
Proportion of plasma samples with detectable concentrations of unmetabolized folic acid. Detection rates at week 6 and week 12 were significantly higher compared to baseline (week 0) in both the 1.1 mg (#) and 5 mg (§) groups.

The limit of detection (LOD) of our affinity chromatography-HPLC assay was 100 pg (0.18 nmol/L). Before supplementation, the proportion of women with a plasma concentration of unmetabolized folic acid that was above the LOD was 0.63 (95% CI, 0.39–0.83) in the 1.1 mg group and 0.68 (95% CI, 0.44–0.86) in the 5 mg group (Figure 7). There was a significant change in the proportion of women with detectable concentrations of folic acid over time in both the 1.1 mg group (Cochran's *Q* = 33.89; df = 3; *P* < 0.001) and the 5 mg group (Cochran's *Q* = 33.69; df = 3; *P* < 0.001). Posthoc comparisons to baseline were statistically significant for week 6 and week 12 (*P* < 0.017) but not week 30 in both groups (1.1 mg: *P* = 0.32, 5 mg: *P* = 0.32; Table 4). Comparing the proportions at each time point, there were no significant differences between the 1.1 mg and 5 mg groups (Fisher's exact test; *P* > 0.99).

In the 1.1 mg group, of the seven women who had *undetectable* plasma concentrations of folic acid at baseline, all had undetectable concentrations of folic acid at week 30; of the 12 women who had *detectable* folic acid at baseline, eleven had detectable concentrations of folic acid at week 30 (kappa = 0.89; 95% CI, 0.68–1.00). A slightly lower level of agreement was observed in the 5 mg group of the six women who had *undetectable* plasma concentrations of folic acid at baseline, five had undetectable concentrations at week 30; of the thirteen women who had detectable folic acid at baseline, ten had detectable concentrations at week 30 (kappa = 0.55; 95% CI, 0.18–0.93).

#### 3.3.3. Plasma Concentrations of Unmetabolized Folic Acid

**Figure 8:**
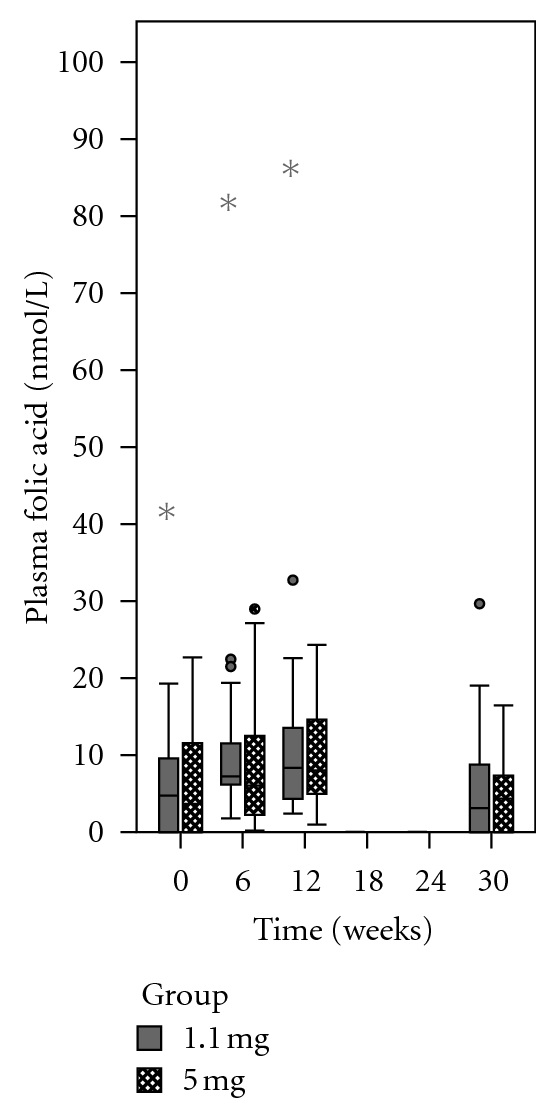
Plasma concentrations of unmetabolized folic acid among women who supplemented with 1.1 mg of folic acid (grey boxes) compared to 5 mg of folic acid (cross-hatched boxes). When analyzed by group, the change in plasma folic acid concentrations was not significant in either the 1.1 mg (*P* = 0.20) or 5 mg (*P* = 0.10) group.

**Figure 9:**
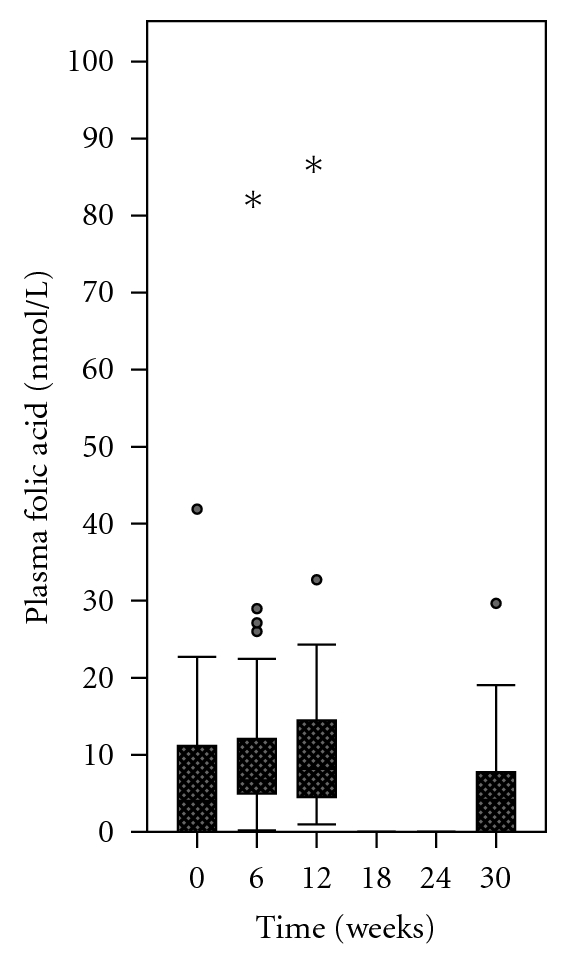
Plasma concentrations of unmetabolized folic acid among women who supplemented with either 1.1 mg or 5 mg of folic acid (all participants combined). When pooled data from both groups were analyzed, there was a significant change in plasma folic acid concentrations (*P* = 0.019). (∗) There was a significant decline from week 12 to week 30 (*P* < 0.05).

The distribution of plasma concentrations of unmetabolized folic acid was not normal, nor was it feasible to transform the data to fit a normal distribution due to the proportion of samples that were below the LOD at baseline and at week 30. The data were, therefore, analyzed using the Friedman test, a nonparametric equivalent to the repeated measures analysis of variance. The data were first analyzed by group (Figures 8 and 9).

At baseline, the median plasma concentration of unmetabolized folic acid was 4.8 nmol/L (undetectable to 41.9 nmol/L) in the 1.1 mg group compared to 3.7 nmol/L (undetectable to 22.7 nmol/L) in the 5 mg group (*z* = 0.06; *P* = 0.95). When analyzed by group, the change in plasma folic acid over 30 weeks of supplementation was not significant in the 1.1 mg group (*χ* ^2^ = 4.71; df = 3; *P* = 0.20) or the 5 mg group (*χ* ^2^ = 6.3; df = 3; *P* = 0.10).

When pooled data from both groups were analyzed, the change in plasma folic acid was found to be significant (*χ* ^2^ = 10.39; df = 3; *P* = 0.019). Posthoc nonparametric multiple comparisons revealed a significant difference between plasma concentrations of unmetabolized folic acid at week 12 and week 30 (*Q* = 4.04; *P* < 0.05).

Comparing plasma folic acid between the two groups at each time point, there were no significant differences (Table 5)

#### 3.3.4. Estimated Dietary Folate Intake

The Block DFE Screener was administered twice during the study—at the baseline study visit to estimate usual folate intake in the six months preceding study participation and at the final study visit to estimate usual folate intake over the course of the study. Differences between groups and over time were evaluated by mixed-model analysis of variance. There was no significant difference between the two groups at baseline or week 30 in dietary folic acid or dietary total folate intake; similarly, there was no significant change in dietary folic acid or dietary total folate intake from baseline to week 30 (Tables 6 and 7). Thus observed changes in unmetabolized folic acid were most likely due to the intervention, as there was no significant change in dietary intake.

## 4. Discussion

The women participating in this study were recruited primarily through advertisements posted in The Hospital for Sick Children and through word-of-mouth. Consistent with other studies conducted through the Motherisk program, the women participating in this study tended to be highly educated and of higher socioeconomic status. All of the women were either enrolled in or had completed postsecondary education; the majority was employed full time. As several studies have shown socioeconomic status, which encompasses education, employment, and income, to be a predictor of folate intake and adequacy [28–31], it was not surprising that most of the women (84%) had usual dietary folate intakes that met or exceeded the EAR for folate (320 *μ*g/day DFE) from diet alone. Approximately three-quarters of the women met or exceeded the RDA of 400 *μ*g/day DFE; none of the women exceeded the UL for folic acid. Estimated dietary total folate intakes (i.e., including natural food folates and folic acid-fortified foods) in our study group were similar to those reported previously among Canadian women of reproductive age [32–34]. Dietary folate intakes remained relatively stable over the course of the study.

None of the participants were found to be folate deficient (serum/plasma folate < 7 nmol/L or RBC folate < 360 nmol/L). Consistent with a recent report on folate status of women of reproductive age in Ontario, about two-thirds of the women had RBC folate concentrations that are associated with a very low risk for NTDs (>906 nmol/L); one-third of the women, therefore, would be at higher-than-baseline risk for NTDs, if they were to become pregnant.

### 4.1. Baseline Concentrations of Unmetabolized Folic Acid

To date, there have been two large population-based studies that examined circulating folic acid concentrations in a country with mandatory folic acid fortification. One study measured unmetabolized folic acid concentrations in plasma samples collected during the sixth examination cycle of the Framingham Offspring Cohort study, which took place between January 1995 and August 1998 [35]; the second study measured folic acid in surplus serum samples collected from NHANES 2001-2002 participants' ≥60 years of age [36].

The prevalence of detectable levels of unmetabolized folic acid in our population at baseline was similar to the prevalence in non-B vitamin users in the Framingham Offspring Cohort examined *after fortification* (67%). In contrast, the median folic acid concentration in our study population was several times higher (3.76 nmol/L compared to 0.50 nmol/L). In the NHANES data set, both the detection rate (38%) and the mean folic acid concentration (1.7 nmol/L; the median was not reported) were lower compared to our population.

The underlying reasons for the apparent discrepancies between detection rates and plasma concentrations are unclear. We used an affinity-HPLC method with electrochemical detection based on the method described by Bagley and Selhub [24], as did the Framingham Offspring Cohort and NHANES studies. The reported detection limits were similar (0.18 nmol/L). However, as reviewed by Bailey and colleagues [36], available studies of unmetabolized folic acid do not demonstrate a relationship between detection rates and LODs, suggesting that the observed discrepancies are more likely due to real differences between the study populations rather than methodological issues. Both the Framingham Offspring Cohort and NHANES studies examined an older population consisting of both men and women, whereas our population consisted of women of reproductive age. The NHANES population is heterogeneous and nationally representative whereas the Framingham Offspring Cohort and our population are more homogenous and self-selected.

Kalmbach and colleagues identified four predictors of “high” folic acid concentrations: dietary folic acid intake, dietary total folate intake, use of B-vitamin supplements, and plasma total folate [35]. We excluded women who reported use of folic acid-containing supplements in the six months preceding study participation and the remaining three measures were not substantially higher in our study population compared to those reported for non-B vitamin users in the Framingham Offspring Cohort. Interestingly, these three measures also did not differ between participants with detectable and undetectable folic acid *within* our population, although the power of this comparison may have been limited by the smaller number of women who had undetectable folic acid (*n* = 13). Another study described a possible “threshold” effect for serum total folate such that the proportion of serum folate as folic acid in samples above 50 nmol/L was found to be significantly higher compared to those below 50 nmol/L [37]. Approximately one-third of the women in this study had a baseline plasma total folate concentration that was greater than 50 nmol/L; however, these thirteen women accounted for almost half of the plasma samples that did not have detectable levels of folic acid. Therefore the higher baseline folic acid concentrations in our population compared to the Framingham Offspring Cohort are not well explained by these factors.

### 4.2. Relationship between Plasma Folic Acid and Other Measures of Folate Status

The majority of studies describing adverse effects of folic acid and/or folate have evaluated exposures in terms of high folic acid intakes or high plasma or serum folate concentrations. Circulating unmetabolized folic acid has only recently gained the attention of researchers, as prior to the development of chromatographic methods for measuring folate in biological samples, available methods (e.g., microbiological assays, folate-binding assays) did not distinguish between folic acid and reduced folates. With the advent of these chromatographic methods, studies have been able to show that dietary folate intakes and blood folate concentrations are predictive of circulating folic acid. To date, however, these relationships have not been studied in a Canadian population

The majority of the women (79%) did *not* have usual dietary folic acid intakes that exceeded the threshold dose for the appearance of unmetabolized folic acid in plasma; none consumed more than 400 *μ*g/day, which has been shown to produce a sustained appearance of folic acid in plasma [38]. That folic acid was detectable in the majority of women in spite of low estimated folic acid intakes suggests that either (a) threshold doses are in fact lower than previously reported or (b) estimated folic acid intakes provided by the Block DFE Screener were not accurate.

The activity of DHFR, which is the rate-limiting step in the conversion of folic acid to 5-CH_3_-H_4_PteGlu, is highly variable. In a study using fresh human liver tissue, DHFR activity was found to vary approximately fivefold among samples [39]. Presumably, individuals with lower levels of DHFR activity would have lower thresholds and individuals with higher levels of DHFR activity could consume and metabolize larger doses of folic acid. Such variation in the metabolism of folic acid could explain why, contrary to our hypothesis, we did not observe a significant correlation between plasma folic acid and dietary folic acid or total folate intake.

In the present study, dietary folic acid and dietary total folate intakes were estimated using the Block DFE Screener, a validated, folate-targeted, semiquantitative FFQ designed to measure usual and customary intake of dietary and supplemental folate. It was designed as an instrument that would rank subjects well according to folate intake and includes the 19 food groups that contributed to 60% of total folate intake in the United States in NHANES 1999-2000 [40]. However, dietary patterns of Canadians and Americans may be quite different and the quality of food composition tables has been called into question [41], which could reduce the validity of this questionnaire in our population and attenuate the correlation with plasma folic acid.

A recent study of pregnant and postpartum women in Canada found that, similar to NHANES 1999-2000 data, grain products were the greatest contributors to dietary folate intake, followed by fruits and vegetables [33]. These groups are well represented in the Block DFE Screener; however, there are items included in the questionnaire that are not major contributors to Canadian intakes, including meal replacement drinks and bars, hot cereals, tortillas, and beer. Conversely, there are major contributors to dietary folate intake among Canadian women that are not represented in the Block DFE Screener, including dairy products and fast foods. The omission of these food groups could result in inaccurate estimates of folic acid intake for women who consume them.

#### 4.2.1. Relationship between Plasma Folic Acid and Blood Total Folate

The relationship between plasma concentration of unmetabolized folic acid and plasma total folate has been evaluated in several studies. Sweeney and colleagues measured plasma folic acid and plasma total folate in fasting plasma samples obtained from women undergoing elective Caesarean section and in nonfasting plasma samples obtained from a random sampling of individuals attending a blood donor clinic [42]. In both populations, plasma folic acid and plasma total folate were significantly correlated, although the strength of the correlation was stronger in fasting samples (*n* = 20; *r* ^2^ = 0.300) compared to nonfasting samples (*n* = 50; *r* ^2^ = 0.110).

In general, studies that found an association between plasma folic acid and plasma or serum total folate included both supplement users and nonusers. As a result, although dietary folic acid intakes were comparable to intakes in our population, mean or median *total* folic acid intakes (i.e., diet and supplements combined) were higher and the ranges of intakes were larger. The restricted range of dietary folic acid intakes in our population might explain the absence of correlation between plasma folic acid and plasma total folate. Because the median plasma total folate concentration in our study was comparable to, if not higher than, mean or median concentrations in previous studies, this further suggests that women in our study were achieving higher plasma folate concentrations as a result of higher intakes of naturally occurring food folates, and not folic acid.

### 4.3. Effect of Folic Acid Supplementation on Circulating Unmetabolized Folic Acid

Current guidelines advise all women who could become pregnant to consume a daily multivitamin containing 0.4 mg to 1 mg of folic acid [43–45]. Women who are at higher risk for having a baby with a NTD are advised to consume a daily multivitamin containing 4 to 5 mg of folic acid, beginning at least three months before conception. Some authorities also recommend the high-dose strategy for women who have a history of poor medication adherence in addition to lifestyle issues that may increase their risk for NTDs [43].

Until recently, however, there was limited data on the pharmacokinetics of the higher dose of folic acid. Nguyen and colleagues were the first to formally investigate the single-dose and steady-state pharmacokinetics of the 5 mg dose of folic acid in women of reproductive age [22, 46]. As the high-dose strategy provides more than ten-times the dosage that appears to saturate both hepatic metabolic capacity and plasma clearance mechanisms, we decided to expand on these findings by examining the effect of daily supplementation with 5 mg compared to 1.1 mg of folic acid on fasting plasma concentrations of unmetabolized folic acid. To the best of our knowledge, this was the first interventional study to evaluate the effects of long-term folic acid supplementation on plasma concentrations of unmetabolized folic acid among women of reproductive age who are also exposed to folic acid fortification.

#### 4.3.1. Supplementation Increases Plasma Concentrations of Unmetabolized Folic Acid

We observed a significant increase in the proportion of women with detectable levels of unmetabolized folic acid over the first 12 weeks of supplementation; concentrations of unmetabolized folic acid also appeared to increase, however, although the overall effect of supplementation was significant, the differences between baseline and week 6 or week 12 were not statistically significant. This suggests that, although there is an effect of supplementation, it is small relative to the natural variation in circulating folic acid concentrations.

There is limited information on the effect of folic acid supplementation on plasma concentrations of unmetabolized folic acid. Bailey and colleagues have presented, in abstract form, preliminary data from a series of small trials evaluating the effects of 10 to 12 weeks of daily supplementation with 0.4 to 5 mg of folic acid in adults in the United States after fortification [47–49]. These studies included both men and women of varying ages and ethnicities. Plasma concentrations of unmetabolized folic acid at baseline were, on average, 0.5 to 0.7 nmol/L. At the lowest dose tested (i.e., 0.4 mg/day), plasma folic acid increased approximately twofold over 12 weeks of supplementation. With higher doses (i.e., 1 mg/day, 2.5 mg/day, or 5 mg/day), plasma folic acid concentrations increased approximately threefold.

Similar to Bailey and colleagues' findings, we also found that the median plasma concentration of unmetabolized folic acid doubled (approximately) over the first 12 weeks of supplementation; this was true in spite of baseline values in our population that were, on average, five-times higher. The discrepancy in baseline values is likely due in part to their exclusion of not only individuals who consumed folic acid supplements in the three months preceding their participation in the study, but also those who reported “significant” consumption of folic acid from dietary sources (e.g., fortified breakfast cereals, energy bars, etc.) (J.E. Ayling, personal communication). There are likely other differences between the study populations with respect to characteristics such as age, sex, and ethnicity—some of which may be associated with differences in folic acid metabolism.

It has been suggested that individuals who have detectable levels of unmetabolized folic acid may represent a subpopulation that has altered folic acid metabolism and responds differently to ingested folic acid [36]. In our study, all of the women had detectable folic acid at some time over the course of supplementation; however, it is interesting to note that almost all of the women who had undetectable folic acid at baseline also had undetectable levels at week 30 and almost all of the women who had detectable folic acid at week 30 also had detectable levels at week 30. It is also interesting to note that, comparing the “detectable” and “undetectable” groups, there was no significant difference in dietary folic acid or total folate intake, suggesting that the women who had detectable folic acid at baseline and throughout the study may represent a “sensitive” group in our population. It would have been interesting to investigate the effects of folic acid supplementation among these women compared to the women who had detectable levels only during the interim study visits, however, our limited sample size precluded such analyses.

If such subpopulations do exist and were more highly represented in our study compared to Bailey and colleagues' studies, this might also explain the differences in baseline concentrations of unmetabolized folic acid and response to supplementation.

#### 4.3.2. Plasma Concentrations of Unmetabolized Folic Acid Do Not Remain Elevated

An unexpected observation was a significant decline in concentrations of unmetabolized folic acid between week 12 and week 30, despite ongoing supplementation and sustained total folate concentrations. In fact, plasma concentrations of unmetabolized folic acid at week 30 were not significantly different from concentrations at baseline. Preliminary data from a study that examined folic acid concentrations before and after six months of folic acid supplementation among women of reproductive age observed only minimal changes in folic acid concentrations with doses up to 4 mg/day [37], which is similar to what we observed in our population. Unfortunately, data were not available (or not collected) at smaller intervals over the course of supplementation, thus it is not known whether unmetabolized folic acid concentrations were significantly higher during the interim, as they were in ours (compared to week 30).

 We first considered the possibility that adherence decreased over the latter half of the study. In a recent study of prenatal multivitamin supplementation in a cohort of Motherisk callers who had either discontinued a previous multivitamin or had yet to start multivitamin supplementation in pregnancy, the most common reasons for discontinuing or not starting supplementation were nausea and vomiting of pregnancy (NVP), difficulty with taking multivitamins, and adverse gastrointestinal events. Women in the present study, however, did not experience NVP (as they were not pregnant) and although approximately one-third of the women reported adverse events, all were mild in nature and did not result in discontinuation of the intervention or withdrawal from the study. In fact, the median rate of adherence approached 90% in each group. We did not obtain week-by-week or month-by-month records of pill intake, thus we cannot exclude the possibility that adherence was higher in the initial weeks of the study before falling off towards the end; however, no decrease was observed in plasma or RBC total folate concentration. This suggests that pill intake occurred at relatively consistent rate over the course of the study and that the decrease in plasma folic acid was not the result of decreased adherence.

One possible mechanism for the observed decline in plasma concentrations of unmetabolized folic acid is upregulation of folic acid metabolism [9, 50]. Kamen and colleagues found that, as compared to human liver cells *in situ*, the expression and activity of DHFR was 100- to 200-times higher in human cell lines *in vitro* and fresh rat liver cells. It was postulated that exposure to high levels of folic acid in culture medium and laboratory rodent chow upregulated DHFR activity in cultured cells and in laboratory animals [51]. Theoretically, a similar process may occur in DHFR at the intestinal mucosa.

DHFR expression is partly controlled by a translational autoregulatory mechanism, where binding of DHFR to its cognate mRNA inhibits translation of the transcript [23]. Binding of H_2_PteGlu to the DHFR-mRNA complex induces a conformational change that releases the mRNA transcript, resulting in resumption of translation and DHFR synthesis. As the first step in the metabolism of folic acid to coenzymatic forms is the reduction of folic acid to H_2_PteGlu, cells exposed to high levels of folic acid would likely accumulate high levels of H_2_PteGlu as well. Thus folic acid, via reduction to H_2_PteGlu, could theoretically upregulate DHFR expression by translational derepression. Further studies are needed to determine whether or not induction of DHFR by folic acid occurs *in vivo* and, if so, to what clinical outcome.

#### 4.3.3. Effect of Dose of Folic Acid Supplementation

Contrary to our original hypothesis, plasma concentrations of unmetabolized folic acid were not significantly higher in the 5 mg group compared to the 1.1 mg group. This is consistent with preliminary data from the series of studies conducted by Bailey and colleagues that found no significant difference in folic acid concentrations achieved over 10 weeks of supplementation with daily doses up to 5 mg (compared to 1 mg) [47–49]. Although we cannot exclude the possibility that individuals consuming a supplement containing 5 mg of folic acid daily will temporarily be exposed to higher amounts of folic acid immediately after dosing, taken together, these data suggest saturation of folic acid uptake and/or retention and the existence of mechanisms that restore and maintain folate homeostasis following ingestion of pharmacological doses of folic acid. Nguyen and colleagues' analysis of plasma and RBC total folate concentrations achieved in this trial of folic acid supplementation were also suggestive of a limiting mechanism, as only a twofold difference in plasma and RBC total folate concentrations was observed despite a fivefold difference in dose [22].

At physiological doses, folic acid is absorbed via carrier-mediated transport involving the PCFT and RFC [7]. Pharmacological doses, however, saturate carrier-mediated transport systems and are likely absorbed primarily via passive diffusion [4]. As passive diffusion is a slower and less efficient means of absorption, this may explain the apparent nonlinearity in steady-state pharmacokinetics of pharmacological doses of folic acid.

Another mechanism may involve downregulation of the intestinal and/or renal transporters that are responsible for folate absorption and reabsorption, respectively. Using the Caco-2 cell line model of the intestinal epithelium and HK-2 cells (proximal renal tubule epithelial cells), Ashokkumar and colleagues observed that carrier-mediated uptake of tritiated folic acid by Caco-2 and HK-2 cells maintained in folate-oversupplemented media was significantly and specifically lower compared to cells maintained in folate-sufficient media [20, 23]. This was accompanied by significantly lower levels of RFC and PCFT protein in both intestinal and renal epithelial cells and of folate receptor protein in renal epithelial cells. This downregulation appeared to be mediated in part via a transcriptional mechanism, as mRNA transcript levels and promoter activity were lower in folate-oversupplemented cells. The reduction in folate receptor protein is of particular interest to this discussion because it is unique among the three proteins studied in that it has a higher affinity for folic acid than it does for reduced folates [52]. At least one-quarter of a 4 mg dose of folic acid is excreted unchanged as a result of exceeding the renal capacity for reabsorption, which is mediated by the folate receptor [53]. This would be exacerbated by a reduction in folate receptor expression and might offer a partial explanation for the absence of significantly higher levels of circulating folic acid in 5 mg group.

As the PCFT and folate receptor are also expressed in peripheral tissues, downregulation of these transporters would theoretically lead to decreased cellular uptake of folic acid. Although this would lead to *higher* concentrations of unmetabolized folic acid in plasma, it would also predict a larger proportion of the dose being available for renal excretion. This may be compounded by saturation of cellular folate pools under conditions of high folate intake; as cellular folate concentrations increase, there is increased competition for FPGS and the marginal formation of polyglutamates decreases [54]. Under these conditions, only a small proportion of folate that enters the cell is retained and the majority is released back into plasma.

In the primary analysis of this trial, Nguyen and colleagues found that women receiving the 5 mg dose of folic acid achieved significantly higher plasma and RBC *total* folate concentrations compared to women receiving the 1.1 mg dose [22]. This, together with our finding that unmetabolized folic acid concentrations were not significantly higher, suggests that the higher total folate concentrations achieved with the 5 mg dose of folic acid constitute reduced, coenzymatic folates. Therefore, upregulation of DHFR, as described in the previous section, may be another mechanism by which exposure to unmetabolized folic acid was regulated among women in the 5 mg group.

It is clear that folic acid supplementation is important for planning and pregnant women. What is not clear, however, is the optimal dose of folic acid needed for the prevention of NTDs and other folate-dependent congenital malformations. Current guidelines advise all women who “could become pregnant” to consume a daily multivitamin providing 0.4 mg to 1 mg of folic acid; a woman deemed to have personal characteristics or health conditions associated with an elevated risk of having a baby with an NTD may be advised to consume a higher dose of folic acid, depending on her contemporaneous folate status. However, there is a lack of research on the pharmacokinetics and safety of high-dose folic acid supplementation.

Folic acid is generally considered to be safe at doses up to 1 mg/day and there is little evidence to show that doses up to 5 mg/day are harmful to healthy adults. In recent years, however, there has been increasing concern that exposure to unmetabolized folic acid, which results from folic acid intakes that overwhelm the body's metabolic capacity, may be associated with adverse effects.

In Canada, legislation mandating fortification of enriched cereal grains with folic acid was introduced in 1998, resulting in universal increases in folic acid intakes and folate concentrations in the blood. In addition to consuming folic acid-fortified foods, many women also consume supplements containing folic acid, thus it is important to develop a better understanding of the relationship between unmetabolized folic acid and dietary and biochemical indicators of folate status and the effect of supplementation.

In this study, we evaluated plasma unmetabolized folic acid in relation to dietary folate intake, blood total folate concentration, and the effect of supplementation among healthy women of reproductive age using plasma samples collected from a randomized trial comparing 30 weeks of supplementation with 1.1 mg or 5 mg of folic acid per day. To the best of our knowledge, this was the first clinical trial that was conducted for the purpose of evaluating the pharmacokinetics of high-dose folic acid supplementation in this population and the data presented herein are the first to describe the folic acid status of women of reproductive age in a folic acid-fortified population.

In this study, we found that unmetabolized folic acid is present at low levels in the majority of women who *do not* consume folic acid supplements but who *do* consume folic acid-fortified foods. Contrary to our original hypothesis, however, there was no significant correlation between plasma folic acid and dietary folic acid or total folate intake or between plasma folic acid and plasma or RBC total folate in samples collected at baseline (i.e., before supplementation). The former may reflect imprecision in our method of dietary assessment and the relatively restricted range of folic acid intakes in our population. On the other hand, the latter suggests that the ability to metabolize folic acid to reduced derivatives is highly variable and that *this* may be a more important determinant of systemic exposure to unmetabolized folic acid.

Upon initiation of supplementation, we observed a significant increase in the proportion of women who had detectable levels of unmetabolized folic acid in fasting plasma samples; however, the increase was not sustained. A similar rise and fall was observed in the concentrations of unmetabolized folic acid over the 30-week supplementation period; however, the increase in plasma folic acid over the first 12 weeks of supplementation did not reach statistical significance. After 30 weeks of supplementation, both the proportion of women with detectable folic acid and concentrations of folic acid returned to levels that were not significantly different compared to baseline. For both measures, there were no significant differences between the women receiving the 1.1 mg dose and those receiving the 5 mg dose. These data suggest that there are homeostatic mechanisms that limit systemic exposure to circulating folic acid, such as downregulation of carrier-mediated transport systems and upregulation of folic acid metabolism.

Taken together with data previously published by our group, it appears that women who supplement daily with 5 mg of folic acid achieve higher plasma and RBC total folate concentrations compared to women who supplement with 1.1 mg/day *without* an apparent increase in exposure to unmetabolized folic acid. This further suggests that the higher plasma and RBC total folate concentrations achieved with the 5 mg dose of folic acid represent reduced, coenzymatic folate; however, additional studies will be needed to confirm.

In summary, this work both corroborates and contradicts current and common views on folic acid metabolism. For instance, hepatic DHFR activity in humans is considered to be highly variable but universally low. The variation in unmetabolized folic acid concentrations before and during supplementation that we observed supports the notion that hepatic metabolic capacity is highly variable; however, for many women in the present study, plasma concentrations of unmetabolized folic acid remained low even though they were consuming 2.5- to 12.5-times the daily dose that was previously shown to produce a sustained appearance of folic acid in plasma. For these women, it would appear that concerns surrounding excessive exposure to unmetabolized folic acid with the 5 mg dose may be unwarranted; thus further consideration could be given to the high-dose folic acid strategy for the primary prevention of NTDs even in the absence of the standard risk factors. On the other hand, until more is known about the safety of exposure to unmetabolized folic acid, alternative approaches to achieving optimally protective folate concentrations could be considered for women who have a lower capacity to handle folic acid. One alternative could be supplementation with levomefolic acid (the calcium salt of 5-CH_3_H_4_PteGlu).

## Figures and Tables

**Table 1:** Composition of study drugs, PregVit and PregVit-Folic5.

Pink (morning) tablet		Blue (evening) tablet	
Vitamin A (as beta-carotene)	2700 IU	Folic acid	
Vitamin B1 (thiamin mononitrate)	3 mg	(PregVit)	1.1 mg
Vitamin B2 (riboflavin)	3.4 mg	(PregVit-Folic5)	5 mg
Vitamin B3 (niacinamide)	20 mg	Vitamin B12 (cyanocobalamin)	12 *μ*g
Vitamin B5 (pantothenate calcium)	5 mg	Vitamin D3 (cholecalciferol)	250 IU
Vitamin B6 (as pyridoxine)	10 mg	Calcium (calcium carbonate)	300 mg
Vitamin E (dL-*α*-tocopheryl acetate)	30 IU		
Copper (cupric oxide)	2 mg		
Iodine (potassium iodine)	0.15 mg		
Iron (ferrous fumarate)	35 mg		
Magnesium (magnesium oxide)	50 mg		
Zinc (zinc oxide)	15 mg		

**Table 2:** Patient characteristics.

	PregVit (1.1 mg folic acid) (*n* = 19)	PregVit-Folic5 (5 mg folic acid) (*n* = 19)	*P* ^a^
Age (years)	33.4 ± 5.5	35.1 ± 7.0	0.40
Weight (kg)	54.5 (45.5–90.9)	61.8 (50.08–6.36)^b^	0.98
Gravidity^c^	0 (0–4)	1 (0–6)	0.86
Ethnicity			0.29
Caucasian	14	14	
Hispanic	1	0	
South Asian	3	1	
Oriental Asian	1	4	
Education			0.78
High school	0	1	
College	6	4	
University	12	12	
Postgraduate	1	2	
Employment			0.73
Student	2	4	
Part time	2	1	
Full time	14	14	
Homemaker	1	0	
Substance use			
Alcohol	11	15	0.29
Cigarettes	0	1	>0.99

^a^ *P* value, as determined by Student's *t*-test, Wilcoxon-Mann-Whitney test, or Fisher's exact test.

^b^Data was missing for one patient (i.e., *n* = 18).

^c^The proportion of women who had been pregnant before was not significantly different between the two groups (9/19 versus 10/19; Fisher's exact test, *P* > 0.99).

**Table 3:** Baseline dietary and biochemical data for participants with detectable or undetectable (i.e., below the LOD) plasma concentrations of unmetabolized folic acid.

	Undetectable (*n* = 13)	Detectable (*n* = 25)	*P* ^a^
Plasma folic acid (nmol/L)	—	8.8 (0.27–41.9)	—
Dietary folic acid (*μ*g/day)	188.5 (56.8–380.0)	191.8 (70.6–395.8)	>0.99
Dietary total folate (*μ*g DFE/day)	477.5 (181.9–849.2)	450.4 (231.9–979.4)	0.52
Plasma total folate (nmol/L)	48.7 (30.3–87.7)	43.7 (27.4–84.8)	0.27
RBC folate (nmol/L)	969.2 (761.0–1777.5)	1018.61 (710.3–2355.1)	0.88

^a^ *P* value, as determined by Wilcoxon-Mann-Whitney test (due to unequal sample sizes).

**Table 4:** Within-group comparisons of the proportion of plasma samples with detectable concentrations of unmetabolized folic acid.

	McNemar's S	df	*P* ^a^
1.1 mg group			
Week 0 versus week 6	7.00	1	0.008
Week 0 versus week 12	7.00	1	0.008
Week 0 versus week 30	1.00	1	0.32
5 mg group			
Week 0 versus week 6	6.00	1	0.014
Week 0 versus week 12	6.00	1	0.014
Week 0 versus week 30	1.00	1	0.32

^a^Critical *P* value = 0.0167 after Bonferroni correction for 3 pair-wise comparisons within each group (0.05 ÷ 3 = 0.0167).

**Table 5:** Between-group comparisons of plasma concentrations of unmetabolized folic acid.

	Plasma folic acid (nmol/L)			
	1.1 mg	5 mg	*z*	*P* ^a^
Week 0	4.76 (ND–41.90)	3.67 (ND–22.71)	0.06	0.95
Week 6	7.23 (1.79–81.92)	6.05 (0.18–28.98)	1.26	0.21
Week 12	8.34 (2.42–86.43)	8.02 (0.97–24.31)	−0.11	0.91
Week 30	3.13 (ND–29.66)	4.35 (ND–16.45)	0.06	0.95

^a^ *P* value, as determined by Wilcoxon-Mann-Whitney test.

**Table 6:** Estimated dietary folic acid intake (*μ*g/day).

	Week 0	Week 30	*P* (week 0 versus week 30)
1.1 mg	221.5 ± 93.9	218.9 ± 93.4	>0.99
5 mg	194.3 ± 82.8	230.1 ± 116.3	0.22

*P* (1.1 mg versus 5 mg)	0.78	0.99	

*There was no significant effect of time (*F*(1, 36) = 1.65; *P* = 0.21), group (*F*(1, 36) = 0.08; *P* = 0.78), or time-group interaction (*F*(1, 36) = 2.19; *P* = 0.15).

**Table 7:** Estimated dietary total folate intake (*μ*g DFE/day).

	Week 0	Week 30	*P* (week 0 versus week 30)
1.1 mg	525.6 ± 192.2	510.5 ± 191.5	0.97
5 mg	491.1 ± 185.6	541.2 ± 248.8	0.64
*P* (1.1 mg versus 5 mg)	0.94	0.97	

*There was no significant effect of time (*F*(1, 36) = 0.43; *P* = 0.52), group (*F*(1, 36) = 0.00; *P* = 0.98), or time-group interaction (*F*(1, 36) = 1.47; *P* = 0.23).
